# Possible Utilization of Distillery Waste in the Carbonization Process

**DOI:** 10.3390/ma15217853

**Published:** 2022-11-07

**Authors:** Jacek Kluska

**Affiliations:** Institute of Fluid Flow Machinery, Polish Academy of Sciences, Fiszera 14, 80-231 Gdańsk, Poland; jkluska@imp.gda.pl

**Keywords:** carbonization, biochar, barbecue, distillery waste

## Abstract

This paper characterizes the carbonization process in terms of the utilization of distillery waste in a laboratory-scale reactor. Due to the increase in market prices of wood and environmental protection laws, biomass waste, including distillery waste, is a potential source for biochar production. An experimental investigation of the carbonization process was carried out for different mixtures of distillery waste and oak sawdust. The obtained results showed that due to the European Standard, biochar from distillery waste could be used for the production of charcoal briquettes for barbecue applications. In addition, biochar from carbonization samples with 66, 50, and 33% distillery waste meet the standards defined by the International Biochar Initiative for HMs content. The analysis of the dynamics of the heating rate showed that adding wood to distillery waste significantly shortens the carbonization process, but this reduces the number of bio-oils produced and its calorific value.

## 1. Introduction

Pyrolysis, according to the combustion process, is an alternative method for biomass and waste disposal, which leads to the conversion of solid waste into solid, liquid, and gas fractions [1,2].

Carbonization, defined as the pyrolysis process focused on the solid fraction, is also widely described in the literature [3]. Due to the application of produced biochar, carbonization is carried out with specific parameters, including heating rate and final temperature.

Nevertheless, due to the increase in wood price and environmental protection, an important aspect is to find the possibility of utilizing different wastes in the carbonization process. Wang et al. [4] and Xu et al. [5] present the characterization of biochar from walnut shells. The influence of the pyrolysis parameters on biochar characteristics has been presented by Almeida et al. [6], who analyzed sugarcane biomass, including bagasse, straw, and treated biomass. In turn, Sahoo et al. [7] showed that biochar from pigeon pea stalk and bamboo meet the standards of biochar production. According to the literature, spent coffee grounds are also an interesting waste in terms of the carbonization process. Andre et al. [8] analyzed biochar from coffee spent in terms of the construction of an energy storage device application. Tangmankongworakoon [9] indicated that biochar from coffee residue might be used as a suitable material for soil amendment.

This work presents the characterization of distillery waste utilization in the carbonization process in terms of barbecue charcoal application due to the European (EN 1860-2) and the International Biochar Initiative Standard.

According to the literature, from 8 to 20 L of stillage on average is produced from every liter of alcohol production [10]. Moreover, the overall production of alcohol reached over 200 billion liters [11]. For this reason, waste management is one of the major problems due to soil pollution and damage to soil and water environments. Mahaly et al. [12], showed the possibility of distillery sludge waste utilization by vermicomposting. Naveen as well as Naveen C., Premalatha [13] present the characteristics of post-methanated distillery effluent using TGA analysis. Dhote et al. [11] analyzed a mixture of distillery waste and coal as a low-cost fuel. The characteristic combustion of distillery waste and coal in a tube furnace with emission analysis has also been presented by Manwatkar et al. [14]. Mohana et al. [15] showed the possibility of anaerobic and aerobic utilization of distillery spent wash. However, there is a lack of papers that characterize the pyrolysis of distillery waste. Dhote et al. [16] characterized the pyrolysis and gasification of distillery sludge and coal in a 3:2 ratio.

This paper presents the carbonization of distillery corn waste pellets with the participation of 100%, 66%, 50%, and 33% by mass. As a well-known material that improves the carbonization process, oak dust was used as an additive. The final temperature was defined at 450 °C due to cooperation with a Barbecue Company. Because of the technical and economic aspects of the carbonization process, in the industrial solution for 2000 kg of wood in a crucible, temperatures of 420–450 °C are applied. The aspect of carbonization at similar temperatures has also been presented by Lima et al. [17], who indicate that 400–500 °C is the optimal range for Amazonian wood carbonization for charcoal production.

The experimental results include the physical and chemical properties of the char, as well as the characteristics of the heating rate of a fixed bed. A valuable part of the work is the characteristics of the carbonization products. This aspect, according to European Biochar Certificate [18], is important because part of the energy (at least 70%) released during the combustion of pyrolysis gases must be used as a heating source or drying biomass. In addition, the combustion characteristics of the produced char were determined to compare the activity and stability of the chars from different blends.

## 2. Materials and Methods

### 2.1. Materials

The characteristics of the distillery waste (corn base distillers) and the wood sample (oak sawdust) are presented in Table 1. The elemental analysis was carried out using a CHNS/O Flash 2000 Analyzer (Thermo Fisher Scientific, USA) and a wavelength-dispersive X-ray fluorescence spectrometer (Bruker Scientific Instruments, Germany).

The heating value was determined using a calorimeter (EkotechLAB, Poland). The volatiles, fixed carbon, and ash content were analyzed according to the EN 1860-2 Standard.

**Figure 1:**
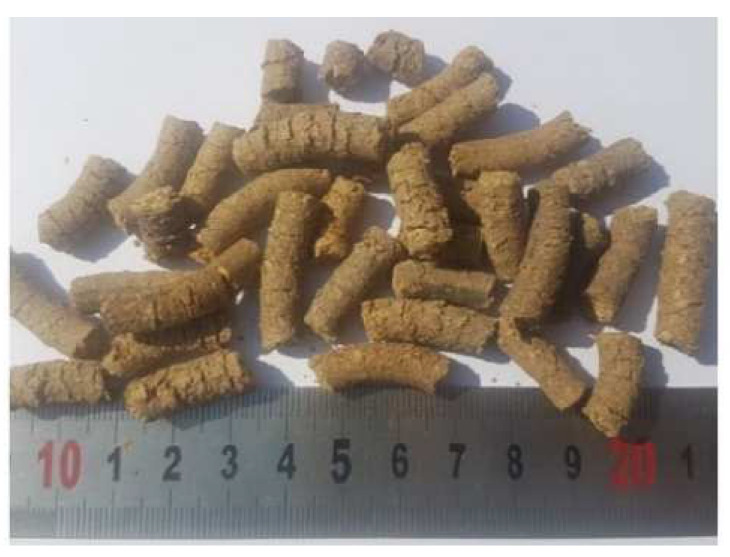
Sample pellets.

The carbonization experiments were carried out using pellets to analyze the dynamic heat transfer of a fixed bed during the carbonization process and to analyze the possibility of improving the carbonization process with a different mixture of distillery corn waste and wood. For this reason, the pellets with a participation of 100%, 66%, 50%, and 33% distillery waste were prepared (Figure 1). According to literature [19], corn-based distillers contain mainly proteins, up to 31%, fats (9–14%), neutral and acid detergent fiber (43–53%), and ash (3–8%).

The wood sample and dried distillery waste were shredded and then mixed in the assumed mass proportion. For the pelletization process, an EkoPal 3kW pellet press, equipped with a flat rotary die and compacting rollers, was used. The pellets consist of cylinder-shaped particles with a diameter of 6 mm and up to 25 mm in length. The variations in the size of the pellets particles are related to the flaw of the pellet mill and the lack of binder.

### 2.2. Carbonization System and Process

**Figure 2:**
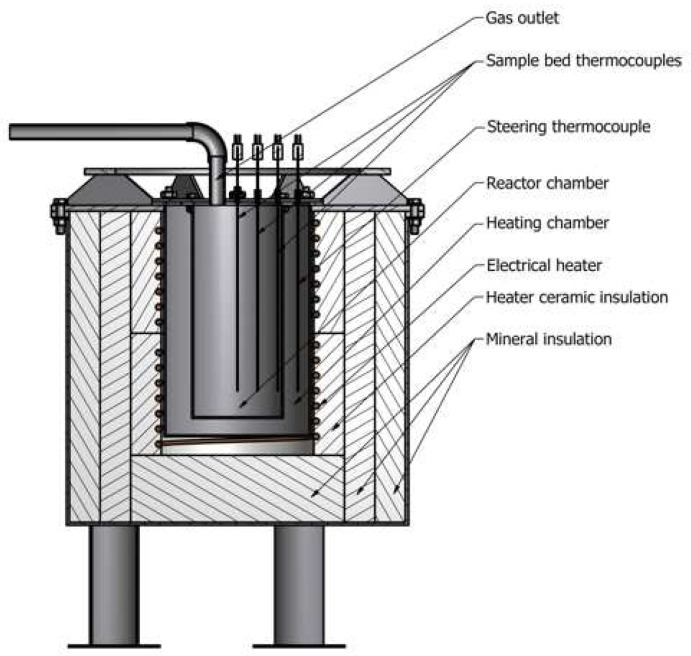
The experimental setup.

The carbonization of distillery waste was carried out in a laboratory-scale batch reactor (Figure 2). The laboratory reactor consists of two thermocouples. The first thermocouple (T_1_) was placed at a distance of 20 mm from the chamber wall and 50 mm from the bottom. The second thermocouple was located in the core of the reactor (T_2_). The reactor was also equipped with an electrical heater with an energy meter to characterize the energy balance of the process.

In each of the experiments, samples with a mass of 2000 g (DW100%), 1800 g (DW66%), 15,000 g (DW50%), and 1300 g (DW33%) were loaded from the top of the reactor. First, the temperature in the heating chamber was set to 450 °C, and then the batch reactor was placed inside the heating chamber. The final temperature for carbonization was set at 450 °C in the core of the fixed bed. Due to maintaining the carbonization process at 450 °C, the heater was turned on automatically using a PID controller when the temperature in the chamber decreased below 450 °C due to heat consumption. When the temperature in the chamber reached 450 °C again, the heater was turned off.

### 2.3. Liquid and Gaseous Products Collection

The experimental investigation included a tar condensation system and a gas sampling system in determining the mass balance and characterization of the liquid products. Due to the analysis of the gas composition, it was first directed to the tar sampling. The sampling of the tar from the carbonization process was carried out using a steel cylinder with isopropanol (capacity 1 L) kept at 0 °C and 3 isopropanol washers kept at −20 °C. After each experiment, the contents of the cylinder and all washers were combined. Due to Karl-Fisher titrator analysis (the water content of the liquid phase) and measuring the initial weight of isopropanol, the number of tars was determined. The calorific value of the tars was determined using a calorimeter (EkotechLAB, Poland) after the drying of sample 2 at a temperature of 105 for 60 min.

The obtained gases were collected in Tedlar bags (1 L) and analyzed every 10 L, measured with a gas meter, to determine the average gas composition. The analysis of gas composition was carried out using a Gas Chromatograph with a thermal conductivity detector (SRI Instruments 310). The calculations of gas caloric was calculated in accordance with Wang et al. [20]:

(1) $$\mathrm{HHV}=Y_{\mathrm{CO}}\cdot {\mathrm{HHV}}_{\mathrm{CO}}+Y_{{\mathrm{CH}}_{4}}\cdot {\mathrm{HHV}}_{{\mathrm{CH}}_{4}}+Y_{H_{2}}\cdot {\mathrm{HHV}}_{H_{2}}+Y_{N_{2}}\cdot {\mathrm{HHV}}_{N_{2}}+Y_{{\mathrm{CO}}_{2}}\cdot {\mathrm{HHV}}_{{\mathrm{CO}}_{2}}$$

where HHV_i_ [MJ/Nm^3^] defines heating value of Y_i_ gas component.

### 2.4. Thermogravimetric Analysis

The characteristics of the thermal degradation and combustion behavior of the char were analyzed by a TA Instruments SDT Q600 Thermogravimetric Analyzer. The sample was heated from 30 to 800 °C at the rate of 10 °C/min. The mass of each sample was 6 mg; the flow rate of air was set at 50 mL/min. According to the literature, the temperature at which the rate of combustion reached 1 wt.%/min was defined as the ignition temperature (T_i_), whereas the burnout temperature (T_b_) was defined as the temperature for which the combustion rate decreased to wt.%/min [21,22]. Moreover, to analyze the combustion process, the S index has been defined according to the following equation [22,23]:

(2) $$S=\frac{{\left(\mathrm{dw}/\mathrm{dt}\right)}_{\max}{\left(\mathrm{dw}/\mathrm{dt}\right)}_{\mathrm{mean}}}{T_{i}^{2}T_{b}},\;$$

where (dw/dt)_ma*x*_ is the maximum and (dw/dt)_mean_ is the average combustion rate. Moreover, the characteristic temperatures and times can be correlated to define ignition (D_i_) and, the burnout (D_f_) index [24]:

(3) $$D_{i}=\frac{{\left(\mathrm{dw}/\mathrm{dt}\right)}_{\max}\;}{t_{i}t_{p}},$$

where t_p_ corresponds to ${\left(\mathrm{dw}/\mathrm{dt}\right)}_{\max}$ [min].

## 3. Results

### 3.1. Heating Rate of Fixed Bed during Carbonization

**Figure 3:**
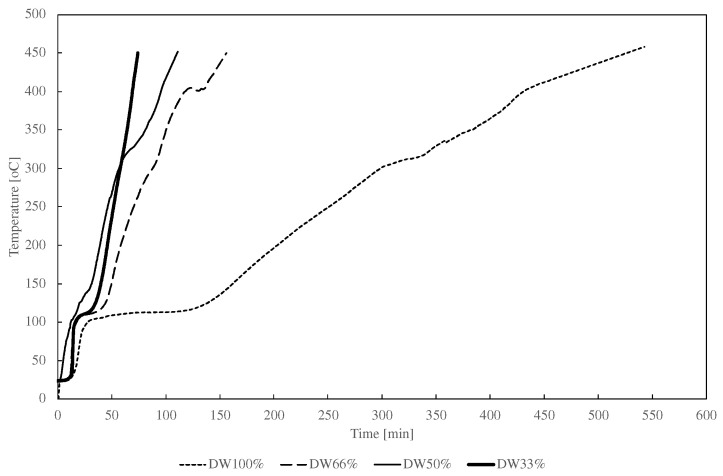
Characteristics of the heating rate of the fixed bed in the reactor for different proportions of distillery waste in the pellets.

Figure 3 presents the characteristics of the heating rate of the pellets with different contents of distillery waste. The experimental results showed that pellets with a content of 100% distillery waste reached 450 °C in 540 min during carbonization. This sample was also characterized by a long plateau associated with the evaporation of moisture. A decrease in the distillery waste content in the pellets to 33% caused an increase in the heating rate and shortened the process to 75 min. A decrease in the content of distillery waste also decreased the power input (recorded with an energy meter) to maintain a constant temperature in the chamber, from 5.3 kWh for DW100% to 1.3 kWh for DW33%.

**Figure 4:**
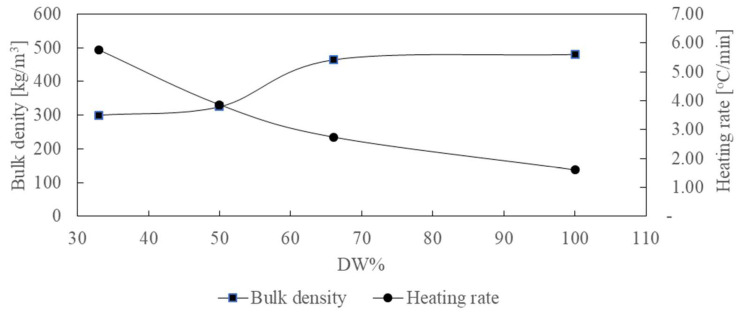
Characteristics of bulk density and heating rate for different proportions of distillery waste in the pellets.

This aspect is also caused by different bulk densities (Figure 4). For the pellets with a content of 100% distillery waste, the bulk density was 480 kg/m^3^, and the heating rate of the bed during carbonization reached 1.6 °C/min. A decrease in the distillery waste content in the pellets to 33% caused a decrease in bulk density to 300 kg/m^3^ and an increase in the heating rate to 5.7 °C/min. The change in the bulk density of the pellets due to the participation of distillery waste also influenced the change in the bulk density of the obtained biochar, which was 480 kg/m^3^, 464 kg/m^3^, 325 kg/m^3^, and 300 kg/m^3^ for DW100%, DW66%, DW50%, and DW33%, respectively.

### 3.2. Characterization of the Carbonization Process

#### 3.2.1. The Effects of Pellets Composition on Char Characteristics

The experimental data showed that the char yield increased slightly with the decrease in distillery waste content in the pellets (Table 2). The carbonization of different samples also indicated that the energy density of the produced biochar reached similar values for all of the samples, about 30 MJ/kg. This may be related to a similar content of fixed carbon. The experimental investigation showed that the change in the proportion of distillery waste in the mixture did not affect the fixed carbon content.

Due to the European Standard [23], for barbecue applications, prepared biochar requires over 75% fixed carbon content for barbecue charcoal and over 65% for charcoal briquettes, with ash contents below 8 and 18%, respectively, which indicates that biochar from distillery waste can be used for the production of charcoal briquettes, while biochar from a mixture of distillery waste and wood meets the standards for the production of charcoal and charcoal briquettes for barbecue applications.

Table 2 also presents the elemental analysis of the obtained biochar. The results show that hydrogen and oxygen (for DW66% and DW33%) significantly decreased, which is caused by the breaking of the weaker bonds within the structure of the char [25,26]. The decrease in oxygen and hydrogen content is also associated with the emission of gaseous and liquid products during the carbonization process [27,28]. According to the literature, the decrease in the hydrogen content is mainly caused by the formation of volatile products from cellulose, mainly anhydrosugars and furanic compounds [29,30], the thermal decomposition of hemicellulose, which leads to the formation of anhydrosugars, ketones, and aldehydes [31]. A decrease in hydrogen content is also associated with lignin decomposition and the emission of phenolic derivatives.

The change in the oxygen content is mainly caused by the emission of carbon monoxide and carbon dioxide [27]. The elemental analysis also indicates that the atomic ratios of H/C reached 0.05–0.07, which meets the requirements specified by International Biochar Initiative for the maximum H/C ratio at 0.7 [18]. It should be noted that the low value of H/C and O/C atomic ratios makes the obtained biochar more aromatic and carbonaceous and leads to a smaller hydrophilic char surface [32,33].

#### 3.2.2. Characteristics of Pyrolysis Gases

Table 3 shows the characteristics of the carbonization products. This aspect is important due to the possibility of using gases for energy purposes.

The conducted analyses showed that a decrease in the distillery waste content in the pellets, with an increase in the wood content, from DW100% to DW33% leads to an increase in the caloric value of pyrolysis gas mixtures from 3.34 MJ/kg for DW100% and increases with an increase in the proportion of wood to 5.45 MJ/kg for DW33%. The analysis of the average gas showed that CO_2_ is the main component, which is caused mainly by the cracking of carboxyl and carbonyl [34]. The carbon monoxide content is related to the cracking of carboxyl and carbonyl groups [35] and decreases from 31% for DW33% to 24% for DW100%. The results of the part release and gas composition are difficult in terms of 66% and 55% of distillation waste participation in the pellets due to the wide temperature range of the thermal decomposition of the distillation waste. The obtained results of the gas composition, due to the limitation of the gas chromatograph, were limited to the main components at the level of 91–95%. The rest of the content may be C_x_H_y_ or other non-detectable compounds.

The characteristics of the obtained bio-oils, considering only the tar content, indicate that the heating value decreased from 35 MJ/kg for distillery waste pellets (DW100%) to 30 MJ/kg for pellets with a 33% proportion of distillery waste. In comparison, Fassinou et al. [36] present that the high heating value (HHV) of vegetable oils (corn, soya, crambe, sunflower, coconut) reaches the value of 38–39 MJ/kg, whereas Santos et al. [35] showed that bio-oils from pyrolysis of sugarcane bagasse and oat hulls at 450 °C reached, respectively, 31 MJ/kg and 33 MJ/kg.

Table 3 also presents the overall energy balance of the liquid and gaseous carbonization products and shows that the caloric value of the produced liquid and gaseous fraction decreased from 17.51 MJ/kg for DW100% to 13.85 for DW330%. Despite the increase in the calorific value of gases caused by the increase in the wood content in the mixture, the amount of produced bio-oils decreased with a simultaneous decrease in the calorific value. Wang et al. [30] indicate that the pyrolysis of proteins isolated from microalgae at 470 °C lead to the production of 38% oils, 25% char, and 37% gas products. Moreover, the obtained oils consisted mainly of aliphatic hydrocarbons, amines, amides, N-heterocyclic compounds, esters, ketones, aldehydes, and nitriles, whereas main compounds from polysaccharides pyrolysis are furans, N-containing compounds, alcohols, carboxylic acid and esters, and sugars.

#### 3.2.3. Influence of Pellets Composition on Elemental Analysis of Char

The obtained results showed that in each sample, the biochar produced contains mainly potassium, calcium, and phosphorus (Table 4). Moreover, the decrease in the distillery waste content in the pellets (DW100%) to 33% content caused an increase in the Fe content from 3.8 to 17.8%. The analysis of heavy metals indicates that the samples obtained from the carbonization pellets with 66, 50, and 33% distillery waste meet the standards defined by the International Biochar Initiative for HMs content in biochar [18].

#### 3.2.4. The Effects of Pellets Composition on the Combustion Characteristics of Char

**Figure 5:**
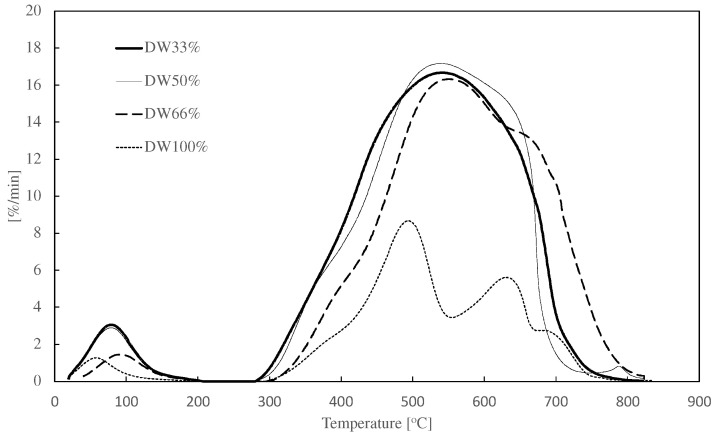
DTG curves of char from carbonization different pellets composition.

Figure 5 presents the DTG curves of the char from the different carbonization pellet compositions. The obtained results indicate that the increases in DW content in the pellet mixture from 33% to 100% led to an increase in ignition temperature (T_i_) from 305 to 343 °C. Moreover, increases in distillery corn waste caused a decrease in the average burning rate from 10.5%/min for DW33% to 4.2%/min for DW100% (Table 5). Wang et al. [37] indicate that this aspect is mainly caused by the combustion of fixed carbon and volatile. A decrease in the fixed carbon and volatile content may lead to a higher average combustion rate.

The experimental investigation and TGA analysis showed that the biochars from the distillery waste are characterized by a very wide, in relation to biochar from corn cobs, cotton stalk, bamboo sawdust, or palm fiber [23,37,38], combustion temperature range.

Table 5 presents the characteristic parameters and indexes that characterize the combustion process. The obtained results of the characteristic temperatures and combustion rate showed that the calculated S index, which defines the combustion reactivity, decreased from 25.6%^2^/(min^2^ °C^3^) for DW33% to 4.5%^2^/(min^2^ °C^3^) for DW100%. It means that an increase in distillery waste content leads to a decrease in the char combustion activity. The increase in distillery waste content from DW33% to DW100% caused a decrease in the ignition index (D_i_) from 0.26 to 0.02%/min^3^. It means that less volatile are degassed from fuel.

Taking into account both indexes, adding wood to the mixture with distillation waste makes combustion more active, efficient and stable [24,39].

The obtained results of the combustion process indicate that there is not much difference between the addition of wood at the level of 50% (DW50%) or 67% (DW33%). Adding only about 34% of wood (DW66%) affects the D index but does not significantly affect the index S and the combustion activity.

## 4. Summary

The results presented in this work indicate the possibility of utilizing distillery waste using the carbonization process. The experimental investigations were carried out for different mixtures of distillery waste and wood. The results of the fixed carbon and ash content indicate that, according to the European Standard, biochar from distillery waste can be used for the production of charcoal briquettes, while biochar from a mixture of distillery waste and wood may be used for the production of charcoal and charcoal briquettes for barbecue applications. The obtained results showed that the thermal decomposition of distillery waste occurs, with regard to wood, in a wide temperature range, with a slightly lower average intensity of mass loss. The analysis of the dynamics of the heating rate showed that adding wood to the distillery waste significantly shortens the carbonization process but reduces the number of bio-oils produced and its calorific value. Taking into account combustion indexes, adding wood to the mixture with distillation waste makes combustion more active, efficient, and stable.

## Figures and Tables

**Table 1:** Proximate and ultimate analysis of distillery waste.

	Distillery Waste	Wood Sample
HHV [MJ/kg]	20.24	17.54
Moisture [wt.%] ^a^	42.2	6.1
Proximate [wt.%] ^b^	5.9	8.4
Volatiles	79.85	81.28
Fixed carbon	18.10	18.38
Ash	2.05	0.34
Ultimate [wt.%] ^b^		
C	47.44	45.00
H	7.49	6.40
O	40.61	47.30
N	4.46	1.30

^a^ as delivered ^b^ db = oven-dry basis.

**Table 2:** Characteristic of carbonization products for different distillery waste content in the pellets.

	DW100	DW66	DW50	DW33
Char yield [wt.%]	33	36	37	37
C_fix_ [wt.%]	75	77	74	78
Volatiles [wt.%]	14	16	18	18
Ash [wt.%]	11	8	7	4
C [wt.%]	49.31	63.62	49.79	59.08
H [wt.%]	3.52	3.49	4.01	3.56
N [wt.%]	5.20	4.20	3.15	4.04
O [wt.%]	41.97	28.69	43.05	33.32
HHV [MJ/kg]	29.25	30.92	30.76	30.86

**Table 3:** Characteristic of pyrolysis gases for different distillery waste content in the pellets.

Composition	DW100	DW66	DW50	DW33
Water [wt.%]	36	33	32	38
Bio-oil [wt.%]	50	47	46	41
HHV [MJ/kg]	35.23	34.70	33.23	30.17
Gases [wt.%]	15	21	22	22
CO [vol.%]	24.10	28.90	29.80	31.70
CO_2_ [vol.%]	59.80	58.66	56.80	51.63
H_2_ [vol.%]	2.90	2.90	3.90	4.70
CH_4_ [vol.%]	4.20	4.67	4.57	7.70
HHV [MJ/kg]	3.34	3.94	4.14	5.45
MJ/kg	17.51	16.67	16.24	13.85
MJ/kg_feedstock_	11.38	7.12	6.90	8.26

**Table 4:** Major nutrient contents of biochars.

		DW100	DW66	DW50	DW33
K	[wt%]	57.9	54.09	41.11	31.66
Ca		10.65	14.37	19.89	24.55
P		17.32	15.41	14.50	12.61
Fe		3.83	5.61	12.33	17.87
S		8.25	7.89	6.04	5.54
Zn		0.99	1.41	1.20	1.58
Mn		0.24	0.67	0.98	1.35
Cu		0.72	0.53	0.51	0.44

**Table 5:** Combustion characteristic parameters of the distillery waste char.

		DW33%	DW50%	DW66%	DW100%
Ti	[°C]	305	313	335	344
T_b_	[°C]	736	709	787	729
t_i_	[min]	6.00	6.11	6.20	16.50
t_max_	[min]	10,64	10.40	10.38	23.64
(dw/dt)_max_	[wt.%/min]	16.66	17.17	16.32	8.66
(dw/dt) _mean_	[wt.%/min]	10.54	10.89	9.76	4.24
S⋅10^7^	[%^2^/(min^2^ °C^3^)]	25.64	26.87	18.05	4.26
D_i_	[%/min^3^]	0.26	0.27	0.25	0.02

## Data Availability

The study did not report any data.
